# Graphene's role in enhancing Fe_3_O_4_ nanofibers: a comparative exploration of room temperature impedance characteristics and EMI shielding performance

**DOI:** 10.1039/d5ra00006h

**Published:** 2025-05-14

**Authors:** Ujala Anwar, Sonia Kiran, Roya Feroze, N. A. Noor, Sadia Nazir, Sohail Mumtaz, Ihab Mohamed Moussa

**Affiliations:** a Institute of Chemical Sciences, Bahauddin Zakariya University Multan 60000 Pakistan; b Physics Department, Comsats University Islamabad Pakistan; c Department of Physics, University of Sargodha 40100 Sargodha Pakistan; d Department of Physics, University of Lahore Lahore 54000 Pakistan; e Department of Chemical and Biological Engineering, Gachon University 1342 Seongnamdaero, Sujeong-gu Seongnam-si 13120 Republic of Korea; f Department of Botany and Microbiology, College of Science, King Saud University P.O. Box 2455 Riyadh 11451 Saudi Arabia

## Abstract

In this work, an electrospinning technique was used for the fabrication of nanofibers to examine the structural, electrical, and EMI shielding characteristics of pure Fe_3_O_4_ and Fe_3_O_4_-graphene (Fe_3_O_4_-Gr) nanofibers, exploring the potential contribution of graphene to the overall performance of Fe_3_O_4_. A consistent fibrous morphology with an average diameter of 62 nm was shown in field emission scanning electron microscopy (FE-SEM) analysis of the Fe_3_O_4_ nanofibers. However, the addition of graphene resulted in few aggregated fibers with an average diameter of 68 nm, which was slightly larger than that of pure Fe_3_O_4_ nanofibers. X-ray diffraction (XRD) pattern confirms that the spinel structure of pure Fe_3_O_4_ was retained in pure Fe_3_O_4_ and Fe_3_O_4_-Gr nanofibers. For both these nanofibers, impedance spectroscopy results showed a single semicircular response, indicating bulk relaxation processes. Fe_3_O_4_-Gr nanofibers exhibited greater bulk resistance at room temperature owing to the increased polarization effects introduced by graphene's conductive pathways. This effect was observed in the modulus plane plots, where Fe_3_O_4_-Gr stored more energy as graphene enabled charge movement and changes in dielectric relaxation. Compared with Fe_3_O_4_, Fe_3_O_4_-Gr nanofibers showed stronger polarization and higher dielectric constants, with two distinct relaxation peaks in the dielectric constant and tangent loss graphs. As per EMI shielding studies, Fe_3_O_4_-Gr nanofibers were better than pure Fe_3_O_4_ in terms of total shielding effectiveness (SE_T_), mainly because graphene's conductive network helped increase the absorption component (SE_A_).

## Abstract 2

X-ray diffraction (XRD) pattern confirms that the spinel structure of pure Fe_3_O_4_ is retained in Fe_3_O_4_ and Fe_3_O_4_-Gr nanofibers. For both nanofibers, impedance spectroscopy showed a single semicircular response, indicating bulk relaxation processes.

## Graphical abstract

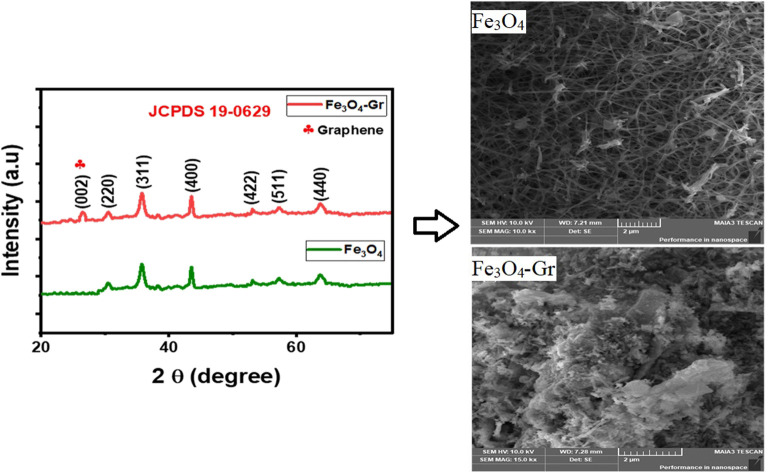

## 1. Introduction

The need for materials with strong electromagnetic interference (EMI) shielding is growing as electronic devices and wireless networks are developing rapidly.^1^ As technology advances, researchers are now focusing on the development of multifunctional, novel composites that are both high performing and sustainable.^2^ Fe_3_O_4_ (magnetite) nanofibers are gaining attention because of their magnetic properties, low toxicity, and electrically adjustable nature.^3^ However, the creation of sophisticated composites is required owing to the limitations of pure Fe_3_O_4_ in terms of impedance characteristics and EMI shielding efficacy at greater frequencies. Because of its mechanical strength, two-dimensional structure, and electrical conductivity, graphene is a promising candidate for modifying pure Fe_3_O_4_.^3^ Graphene can significantly increase the effectiveness of electromagnetic radiation shielding in Fe_3_O_4_ nanofibers while improving their impedance characteristics.^4^ The combined effect of graphene's conductive network and Fe_3_O_4_'s magnetic properties can create a synergistic interaction that enables new approaches to control the dielectric and magnetic responses of this nanofiber material, especially at room temperature, in which several commercial and industrial applications occur.

Nanofiber materials with multifunctionality can be easily produced through electrospinning technique by combining distinct nano fillers with distinct electrical and physical characteristics in order to provide different capabilities to the polymer solutions.^5^ In addition, these materials are found to be viable in several applications, including microwave absorbers, electromagnetic devices, stable dielectric constant and lowest tangent loss which can be beneficial from the use of multifunctional nanofiber materials with electrical as well as magnetic characteristics.^6^ Materials with both magnetically permeable and electrically conductive substances are required to offer electromagnetic characteristics.^7^ Fe_3_O_4_ is one of these substances, and hence, it is useful as a magnetic filler. Fe_3_O_4_ can be used in medical applications, such as in drug delivery and therapy and in microwave absorption structures and dampers when combined with a polymer matrix.^8^ Alternatively, electrical conductivity can be achieved by employing graphene or other electrically conductive polymers.^9^ Previous studies have explored the addition of magnetic nano-fillers, such as conductive polymers, to the matrix of electrically conducting polymers.^10^ We select graphene, which results in enhanced electrical conductivity. It is possible to create magnetic multifunctional nanofiber materials by dissolving the Fe_3_O_4_ precursor in graphene and then carbonizing and electrospinning the mixture to create magnetic composites.^11^

Although Fe_3_O_4_ has significant magnetic ordering, it is a ferrimagnetic material, resulting in improvements in its capacity to absorb electromagnetic radiation. It can also improve the magnetic loss mechanisms, which are essential for EMI shielding, because of its magnetic characteristics.^12^ While Fe_3_O_4_ exhibits moderate conductivity and can influence the dielectric and magnetic properties of composites, it is not as conductive as graphene,.^13^ This helps modulate the impedance properties of the material. The frequency-dependent dielectric properties of Fe_3_O_4_ help adjust the impedance of the nanofiber material at various frequencies. Fe_3_O_4_ improves the electromagnetic radiation shielding efficacy by absorbing instead of reflecting electromagnetic radiation owing to the combination of dielectric and magnetic losses.^14^ Fe_3_O_4_ can be easily combined with graphene and MoS_2_ to create a stable nanofiber material. These nanofiber materials can exhibit synergistic effects that lead to better impedance and EMI shielding qualities when compared to the individual components.^15^

The sp^2^-hybridized carbon atoms that make up the 2D honeycomb pattern of graphene provide excellent electrical conductivity. Because of these properties, it is ideal to enhance the conductive network in nanofiber materials.^16^ By combining with other materials, the higher conductivity can affect the impedance and electromagnetic interference (EMI) shielding capabilities in addition to facilitating effective charge transport.^17^ It is one of the hardest materials that has ever been tested and is renowned for its exceptional mechanical strength and flexibility.^18^ This improves the structural stability of the nanofiber material, particularly for nanofibers. Owing to its enormous surface area (up to 2630 m^2^ g^−1^ for single-layer graphene), graphene can boost the performance of material structures by promoting enhanced interactions with other nanomaterials, such as Fe_3_O_4_.^19^ Because of its high thermal conductivity (up to 5000 W mK^−1^), graphene is a useful material for high-frequency electromagnetic interference applications because it helps disperse heat.^20^ Graphene is a great material for EMI shielding because of its conductive and reflective qualities. High-frequency bands, such as the X-band and Ku-band, shield effectively by reflecting and absorbing electromagnetic waves.

This work investigates the relative impacts of graphene incorporation on Fe_3_O_4_ nanofiber impedance (dielectric constant, tangent loss and ac conductivity) and EMI shielding performance. This work aims to provide deeper insights into the ways in which conductive graphene enhances the electromagnetic behavior of Fe_3_O_4_ nanofiber-based materials by examining their electrical properties at room temperature and their EMI shielding performance within the X-band frequency range. This could lead to the development of new lightweight EMI shielding solutions with high performances. This nanofiber material can change the landscape of materials for wireless networks, next-generation electronics, and other applications.

## 2. Materials and methods

### 2.1. Materials

In this study, commercial sources provided materials and chemicals. Polyvinylpyrrolidone (PVP, molecular weight: 1 300 000), iron(iii) nitrate nonahydrate, *N*,*N*-dimethylformamide (DMF), and graphene are the materials employed in the production of Fe_3_O_4_ nanofibers.

### 2.2. Preparation of Fe_3_O_4_ nanofibers

**Fig. 1:**
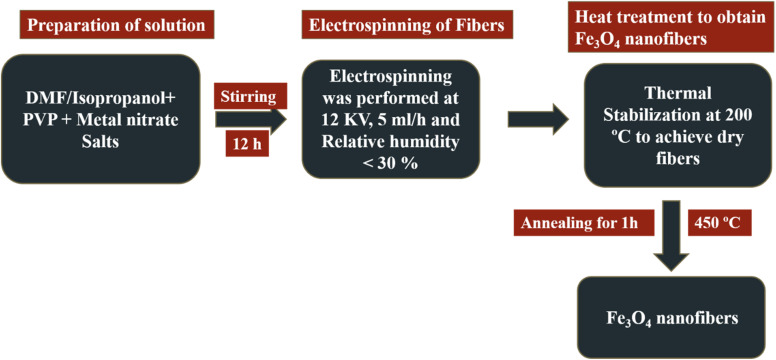
Pictorial representation of the synthesis of pure Fe_3_O_4_ nanofibers.

To prepare the Fe_3_O_4_ nanofibers, an electrospinning solution was obtained by dissolving 10 wt% of polyvinylpyrrolidone (PVP) in 40 mL of *N*,*N*-dimethylformamide and 60 mL of isopropanol at room temperature. A transparent solution was obtained after stirring for 1 h by adding 12% by weight. Iron nitrate was added to the prepared solution. The solution was mixed for 12 h, then put into a 10 mL syringe and run through a 19-gauge needle multi-needle spinneret. The distance between the needle tips and an aluminum foil acquisition plate is 9 cm. To perform electrospinning, a 10 kV voltage was applied, the solution feed rate was 4 mL h^−1^, and the relative humidity was rigorously controlled to be less than 25%. When there is a significant voltage differential between the conducting plate and the needle tip, the electrically charged solution spreads into the needle tip. Because of the strong electric field, the lengthy droplet is ejected in the jet and directed towards a conductive collection plate with an opposite charge. Charged fibers accumulate on the conductive plate as a result of the solvent gradually evaporating as the jet moves along its path. For 1 h, the electrospun fibers affixed to the collection plate are baked at 200 °C to ensure full drying. Fe_3_O_4_ nanofibers are then produced by annealing the nanofibers for 2 h at 450 °C, with a temperature ramping rate of 3 °C per minute.^5^ The schematic flow sheet for the electrospinning process is shown in Fig. 1.

### 2.3. Preparation of Fe_3_O_4_-Gr nanofibers

**Fig. 2:**
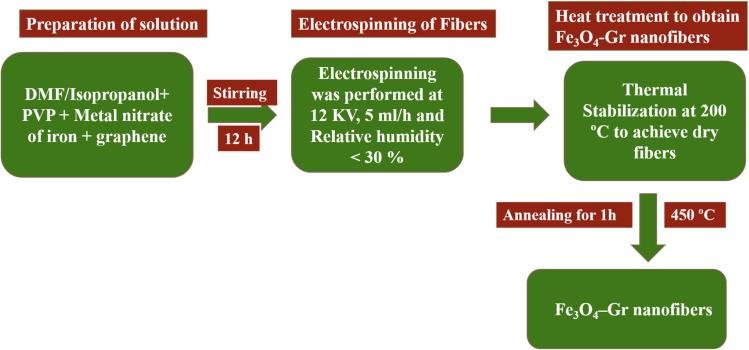
Pictorial representation of the synthesis process of Fe_3_O_4_-Gr nanofibers.

To prepare Fe_3_O_4_-Gr nanofibers, an electrospinning solution was prepared at room temperature. 8 wt% of polyvinylpyrrolidone was dissolved in 40 mL of *N*-dimethylformamide and 60 mL of isopropanol. The mixture was stirred for an hour using a magnetic stirrer. After adding iron nitrate (12 wt%) and graphene (0.8%) to the prepared solution, a blackish-brown solution was formed. Fe_3_O_4_-Gr nanofibers were prepared by applying the electrospinning technique and repeating the above-discussed process after mixing for 12 hours. Fig. 2 displays the electrospinning process flow sheet diagram for the fabrication of Fe_3_O_4_-Gr nanofibers.

The crystallite size and phase composition of the Fe_3_O_4_ nanofibers were determined using an X-ray diffractometer with CuKα radiation, with a step size of 0.02° and a scanning speed of 4 min^−1^ over the range of 15°–80°. The microstructure and morphology were examined using field emission scanning electron microscopy (FE-SEM). Temperature-dependent impedance spectroscopy was conducted using a dielectric analyzer, with data acquisition and analysis carried out using WINDETA software. The impedance measurements were performed on the pelletized samples. Electromagnetic shielding (EMI) properties were evaluated using a vector network analyzer, covering a frequency ranging from 10 MHz to 40 GHz. To assess electromagnetic characteristics and microwave absorption, the synthesized materials were finely ground, homogeneously mixed with paraffin in a 1 : 1 mass ratio, and molded into slabs of 22.5 mm × 10.1 mm for testing.

## 3. Results and discussions

**Fig. 3:**
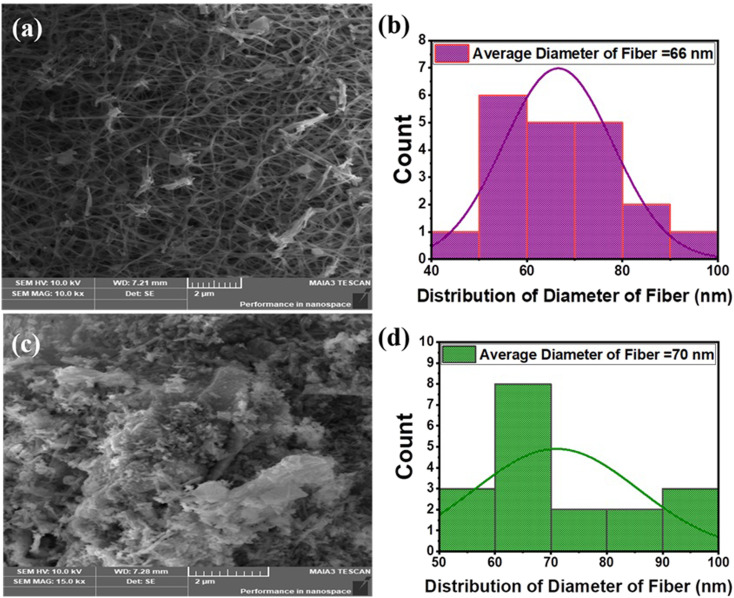
(a and c) FE-SEM images of pure Fe_3_O_4_ and ultra-thin flakes of Fe_3_O_4_-Gr nanofibers at 30 k× magnification and (b and d) average diameter of the fibers.

The unique morphological features of pure Fe_3_O_4_ and Fe_3_O_4_-Gr nanofibers, which have a major impact on their functional properties, are strongly demonstrated by the FE-SEM images of these nanofiber materials. Graphene sheets normally appear as wrinkled, thin, or layered regions dispersed around or between the fibers, while Fe_3_O_4_ nanofibers typically appear as elongated, interwoven fibrous formations. The pure Fe_3_O_4_ nanofibers have a distinct fibrous shape with uniform, continuous fibers, as shown in Fig. 3(a), which have an average diameter of about 62 nm calculated using Image J software, as shown in Fig. 3(b). These nanofibers are useful for shielding against electromagnetic interference (EMI) and storing energy because of their fine fibrous structure, which also increases surface area and promotes effective charge transport. However, the FE-SEM study of Fe_3_O_4_-Gr nanofibers shows a distinct morphological pattern, with tiny aggregated fibers, as illustrated in Fig. 3(c), showing an average diameter of 68 nm, as shown in Fig. 3(d). Because of the integration of graphene, which can cause changes in the dispersion and alignment of Fe_3_O_4_ nanofibers, the aggregation of fibers suggests a more complex interaction between the Fe_3_O_4_ nanofibers and graphene components during synthesis.^21^ Because of its high surface smoothness and thinness, graphene can be difficult to discern in FE-SEM. It may resemble folded sheets linked to nanofibers or be slightly translucent. Possibly in areas with less contrast, graphene surrounds the Fe_3_O_4_ nanofibers as extremely thin flakes or coating layers. This aggregation produces distinct structural characteristics that can improve the overall mechanical and electrical properties of nanofiber materials; however, it also results in a slightly larger average diameter than pure Fe_3_O_4_ nanofibers.

In addition to altering the fiber form, the addition of graphene increases the electrical conductivity of the Fe_3_O_4_-Gr nanofiber. Better interfacial contacts between the Fe_3_O_4_ nanofibers and graphene phases may be facilitated by aggregation, leading to enhanced electromagnetic performance and electroactive charge carrier mobility.^22^ The elongated aggregated morphology of Fe_3_O_4_-Gr nanofibers adds extra complexity that can be used to optimize the material functional properties of nanofibers, even though the fibrous structure of Fe_3_O_4_-Gr nanofibers provides a strong platform for shielding applications. This comparative morphological research highlights the importance of customizing nanostructures to fulfill certain functional needs by emphasizing the crucial role that fiber structure plays in shaping the performance characteristics of these nanofibers.

**Fig. 4:**
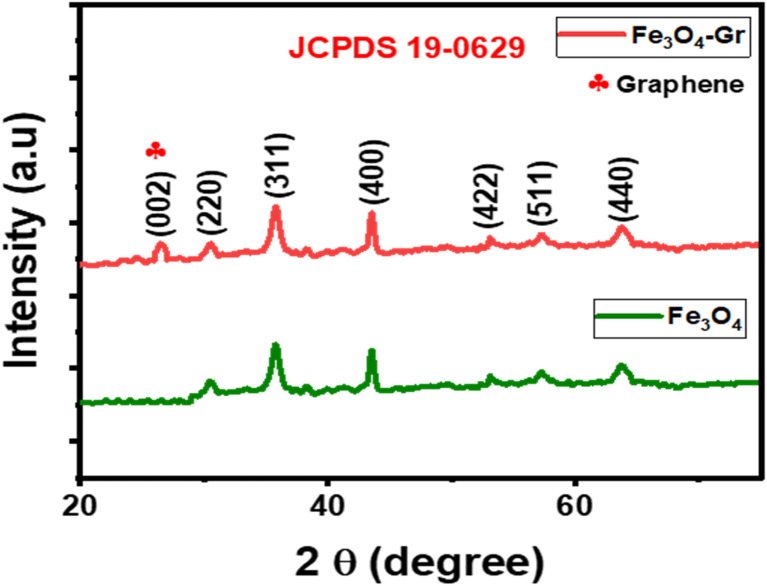
XRD patterns of Fe_3_O_4_ and Fe_3_O_4_-Gr nanofibers.

The XRD patterns of the Fe_3_O_4_ and Fe_3_O_4_-Gr nanofibers provide crucial structural information that highlights the impact of graphene on the crystalline properties of nanofiber materials. The diffraction peaks for Fe_3_O_4_ nanofibers are located at 2*θ* values of 30.1°, 35.4°, 43.1°, 53.4°, 57.0°, and 62.6°, which correspond to the (220), (311), (400), (422), (511), and (440) planes, as shown in Fig. 4.^21^ JCPDS card no. 19-0629 confirms that these peak positions correspond with the distinctive spinel structure of magnetite (Fe_3_O_4_), suggesting a clearly defined cubic crystalline structure. The sharpness and intensity of these peaks indicate the high crystallinity of Fe_3_O_4_ nanofibers, where the octahedral and tetrahedral sites of the spinel lattice contain a regular arrangement of Fe^3+^ and Fe^2+^ ions, which gives rise to the magnetic properties of the material. However, all the distinctive Fe_3_O_4_ peaks are present in the XRD pattern of Fe_3_O_4_-Gr nanofibers. Furthermore, the presence of few-layer graphene is indicated by the observation of a faint peak at around 26.5°, which corresponds to the (002) plane of graphene. Although the major peaks of Fe_3_O_4_ remain aligned with the spinel structure, the addition of graphene affects the lattice properties and crystallinity of the material. The addition of a conductive network and decreased crystallinity caused by the graphene present in the nanofiber material results in novel structural dynamics that improve the material's overall qualities, particularly its mechanical strength and electrical conductivity.

**Fig. 5:**
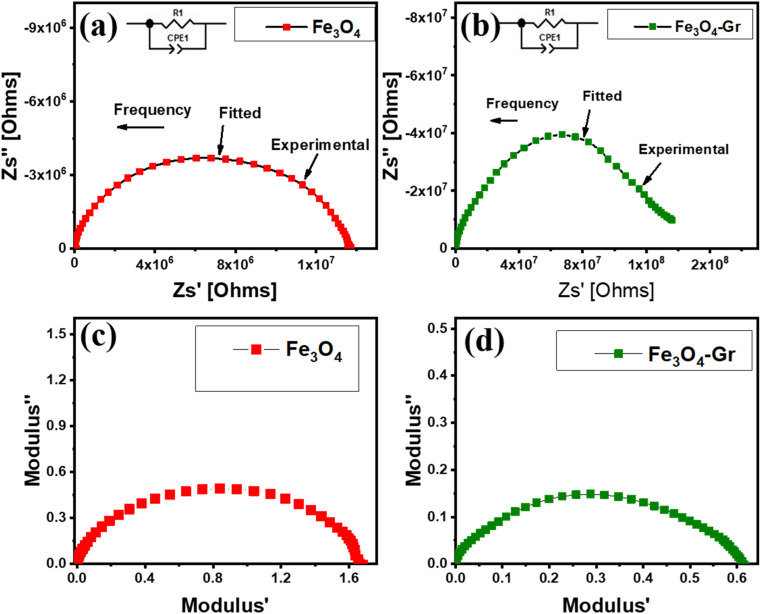
(a and b) Impedance plane plots and (c and d) modulus plots for Fe_3_O_4_ and Fe_3_O_4_-Gr nanofibers at room temperature.

To understand the conduction mechanism of Fe_3_O_4_ nanofibers, impedance spectroscopy (IS) is employed. To better comprehend impedance plane plots, the impedance data are interpreted using Zview software with an analogous circuit model (R_1_Q_1_), as shown in the inset in Fig. 5(a) and (b). The electrical behaviors of pure Fe_3_O_4_ and Fe_3_O_4_-Gr nanofibers at room temperature can be better understood by examining the impedance plane plots, which show the imaginary component of impedance against its real part. A single semicircle that dominates in Fig. 5(a and b) for both materials indicates bulk relaxation in the frequency ranging from 1 Hz to 10^7^ Hz. In Fe_3_O_4_ nanofibers, the semicircle is equivalent to a bulk resistance of about 10^7^ ohms, suggesting that the intrinsic dielectric and magnetic characteristics of Fe_3_O_4_ control the flow of charge within the material. Owing to the magnetic properties of Fe_3_O_4_, which are driven by relaxation processes where localized charge carriers contribute to resistive losses, the relatively lower resistance and smaller semicircle diameter imply moderate charge mobility. The limited conductivity of Fe_3_O_4_ causes charge carriers to encounter strong obstacles in their path, which causes them to dissipate more energy and relax more slowly.^23^ Graphene is added to Fe_3_O_4_-Gr nanofibers, showing a larger semicircle diameter and a greater bulk resistance of around 10^8^ ohms, as shown in Fig. 5(b). Although graphene is known for its conductivity, this greater resistance may appear paradoxical at first, but it represents the bulk relaxation dynamics of the nanofiber material.^24^ Graphene plays two roles in the system: first, it enhances local charge mobility by providing highly conductive routes; second, in the context of bulk relaxation, it intensifies polarization effects and interfacial interactions between Fe_3_O_4_ nanofibers and graphene. Larger semicircles in the impedance plot result from these improved polarization mechanisms, increasing the resistance associated with bulk charge storage and release processes while boosting the overall dielectric response of the nanofiber materials.

Understanding the connection between Fe_3_O_4_ and graphene is essential to comprehending these electrical characteristics. Graphene increases capacitive sensitivity by creating interconnected conductive networks, whereas the magnetic properties of Fe_3_O_4_ drive bulk relaxation and restrict electroactive charge mobility. However, as electroactive charge carriers interact with interfaces and graphene layers in the bulk impedance regime, these interconnected conductive networks lead to increased polarization resistance at room temperature. Therefore, the combined effect of graphene's charge storage influence and Fe_3_O_4_'s magnetic losses results in larger semicircles and higher resistance in Fe_3_O_4_-Gr nanofibers, where the nanofiber material shows both improved bulk relaxation resistance and improved dielectric behavior as a function of frequency. By balancing conductivity and polarization, this interaction transforms Fe_3_O_4_-Gr into a more complicated system and shows how graphene's function goes beyond enhancing conductivity to affect total impedance behavior at both the microstructural and bulk levels.^25^ For pure Fe_3_O_4_ and Fe_3_O_4_-Gr nanofibers at room temperature, the modulus plane plots show the imaginary portion of the modulus against the real part, offering insight into their dielectric relaxation processes, as shown in Fig. 5(c) and (d). For all frequencies between 1 Hz and 10^7^ Hz, a single semicircle is visible in both cases, signifying the bulk relaxation process. However, significant variations in the capacities of the two materials for energy storage and dissipation are observed in the characteristics of the semicircles. A comparatively simple dielectric response dominated by bulk relaxation processes is suggested by the single semicircle for pure Fe_3_O_4_ nanofibers at room temperature. Its intrinsic magnetic characteristics control the dipole alignment and polarization within the Fe_3_O_4_ matrix, which is the primary mechanism for the material's dielectric relaxation, as indicated by the size and relaxation peak position of the semicircle. Pure Fe_3_O_4_ has a limited dielectric constant and slower relaxation dynamics, which are reflected in the semicircle's moderate size.^26^ This is because the real part of the modulus, which represents the material's capacity to store energy, and the imaginary part, which is related to energy loss through dielectric relaxation, are balanced.

In comparison, the Fe_3_O_4_-Gr nanofibers exhibit a single semicircle that is similar but somewhat different, indicating the impact of graphene. Because of its large surface area and conductive pathways, graphene improves the nanofiber material's capacitive behavior and increases the real component of the modulus, which is associated with improved energy storage capacities. Simultaneously, there is a shift in the imaginary component of the modulus, which indicates the energy dissipation of the material. This is because the addition of graphene results in more effective charge redistribution and stronger polarization effects inside the nanofiber material. Consequently, the semicircle becomes larger or slightly displaced, indicating that the dielectric relaxation of the Fe_3_O_4_-Gr nanofibers is higher than that of pure Fe_3_O_4_. The relationship between Fe_3_O_4_ and graphene in this context is key to understanding their electrical properties. Although Fe_3_O_4_ contributes primarily to its magnetic and dielectric response, the addition of graphene significantly improves the ability of the nanofiber material to store and release electrical energy. Graphene's conductive nature provides efficient pathways for charge transport and enhances interfacial polarization, thereby increasing the real modulus and enabling faster relaxation dynamics as a function of frequency at room temperature. This synergistic interaction between Fe_3_O_4_'s magnetic properties and graphene's conductive network results in a nanofiber material with improved dielectric properties and enhanced energy dissipation control, as evidenced by the distinctive behavior in the modulus plane plots.

**Fig. 6:**
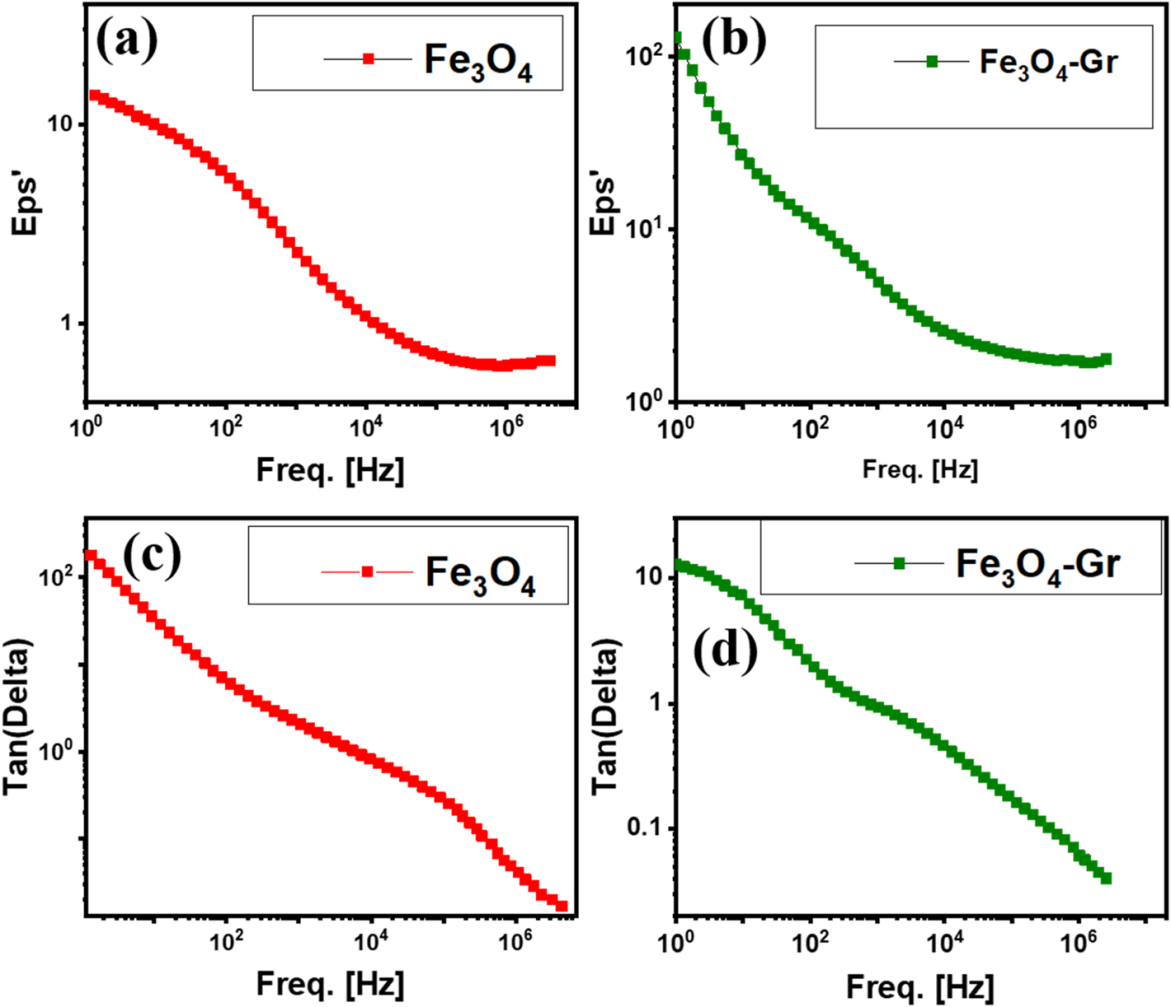
(a and b) Frequency-dependent dielectric constant (*ε*) and (c and d) tangent loss (tan *δ*) for Fe_3_O_4_ and Fe_3_O_4_-Gr nanofibers at room temperature.

The frequency-dependent behavior of the dielectric constant in pure Fe_3_O_4_ and Fe_3_O_4_-Gr nanofibers is characteristic of the capacity of the nanofiber materials to store electrical energy in response to an applied electric field, as shown in Fig. 6(a) and (b). Two relaxation peaks are observed for both nanofiber materials, suggesting the presence of several polarization mechanisms operating in various frequency ranges. The dielectric constant of the Fe_3_O_4_-Gr nanofibers, however, is higher than that of the pure Fe_3_O_4_ nanofibers, reaching values of 10^2^, as shown in Fig. 6(b), at a higher frequency domain. The inclusion of graphene within the nanofiber material, which provides interconnected conductive channels that speed up charge transport and dipole orientation, is responsible for this noticeable improvement in the polarization response.^27^ The magnetic and dielectric characteristics of pure Fe_3_O_4_ lead to polarization at the grain boundaries, and interfacial polarization dominates the dielectric response in pure Fe_3_O_4_ nanofibers. The dielectric constant gradually decreases as the frequency increases, reflecting the incapacity of the dipoles to align with the quickly fluctuating electric field, leading to a moderate energy storage capacity at room temperature.^28^ The enormous surface area and high conductivity of graphene, however, result in a greatly improved dielectric response in the Fe_3_O_4_-Gr nanofiber material, as shown in Fig. 6(b). At the Fe_3_O_4_-Gr interfaces, graphene adds extra interfacial polarization, which increases the dielectric constant at lower frequencies where polarization mechanisms can react to applied fields to the fullest.

Two different loss peaks, one at a low frequency and the other at a high frequency, are shown in the frequency-dependent fluctuation of tangent loss for pure Fe_3_O_4_ and Fe_3_O_4_-Gr nanofibers, as shown in Fig. 6(c) and (d). These peaks are associated with relaxation processes where dielectric losses cause energy to be lost as heat at room temperature.^29^ The lowest tangent loss, however, is shown by both materials between these peaks, suggesting effective energy storage with low dissipation across a wide frequency range (1 Hz–10 MHz). Electroactive charge carrier relaxation and interfacial polarization are the main causes of the larger tangent loss at low frequencies, while the challenge of dipole alignment during fast oscillations is the reason for the peak at higher frequencies. The explanation for these improved dielectric characteristics depends critically on the connection between Fe_3_O_4_ and graphene. Graphene's conductive network helps to reduce dielectric losses and enable a higher dielectric constant in Fe_3_O_4_-Gr nanofibers by improving the overall polarization response and helping to dissipate charge carriers more efficiently. When combined with graphene, Fe_3_O_4_ can achieve greater dielectric performance by accelerating dipole alignment and improving electroactive charge mobility, while pure Fe_3_O_4_ depends on its magnetic characteristics for polarization. Fe_3_O_4_-Gr nanofibers possess superior dielectric storage properties owing to the synergy between graphene's electrical conductivity and Fe_3_O_4_'s magnetic losses.^30^

**Fig. 7:**
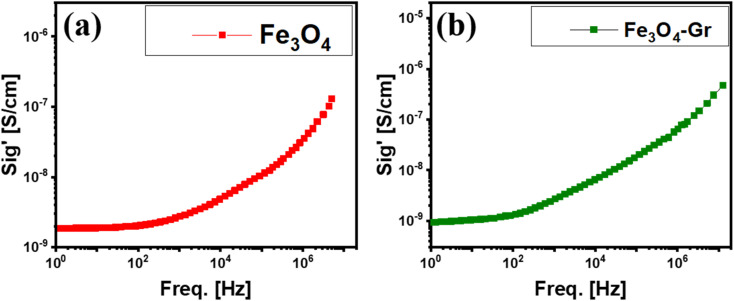
(a and b) AC conductivity (*σ*) for Fe_3_O_4_ and Fe_3_O_4_-Gr nanofibers at room temperature.

The conductivity performance can be described using the formula *σ*_*t*_ = *σ*_dc_ + *Aω*^*s*^, where *σ*_*t*_ is the AC conductivity, *ω* is the angular frequency (2π*f*), *A* is the polarizability factor and “*s*” is the temperature-based exponent.^31^ There are notable differences in the electrical properties of pure Fe_3_O_4_ and Fe_3_O_4_-Gr nanofibers when compared to room temperature, especially when it comes to how they conduct AC. Ohmic conduction dominates charge transfer at lower frequencies, as both materials show frequency-independent DC conductivity, as shown in Fig. 7(a) and (b). Pure Fe_3_O_4_ nanofibers are mostly dependent on their magnetic characteristics in this area, where concentrated charge carriers contribute to their comparatively low conductivity.^32^ Conversely, the Fe_3_O_4_-Gr nanofibers, which also show frequency-independent behavior at low frequencies at ambient temperature, benefit from graphene. The nanofiber material has a higher baseline conductivity than pure Fe_3_O_4_ nanofibers because graphene's highly conductive, linked network allows for more efficient charge transport even in the DC region.^33^ The frequency-dependent AC conductivity takes center phase as the frequency increases, and the conductivities of both pure Fe_3_O_4_ and Fe_3_O_4_-Gr nanofibers exhibit increasing trends, reaching values on the order of 10^−7^ S cm^−1^ at higher frequencies of approximately 10^6^ Hz at room temperature. This increase for Fe_3_O_4_ nanofibers reflects the intrinsic polarization mechanisms of the material, where displacement currents and charge hopping become more prominent at higher frequencies. However, in comparison with the Fe_3_O_4_-Gr nanofibers, the improvement in conductivity is slightly moderate as a function of frequency. When graphene is added, AC conductivity increases more rapidly because of its remarkable electron mobility, which facilitates more effective charge transfer, particularly at higher frequencies at room temperature. The conductive channels created by graphene improve electroactive charge carrier hopping and make it easier for dipoles to align in response to an alternating electric field, which improves the AC conductivity performance of the nanofiber material.

The interplay between Fe_3_O_4_ nanofibers and graphene in the electrical behavior of the nanofiber material is demonstrated by this comparative investigation. Owing to its magnetic and dielectric characteristics, pure Fe_3_O_4_ shows only slight improvements in conductivity; however, the addition of graphene enhances this effect, especially at higher frequencies, where AC conductivity predominates as a function of frequency. Owing to graphene's conductive properties, electroactive charge hopping can occur more quickly and efficiently by lowering energy barriers to charge flow. Because of their improved electrical performance, Fe_3_O_4_-Gr nanofibers are a better option for high-frequency operating applications where conductivity and charge mobility are essential. The conductive network of graphene and the magnetic response of Fe_3_O_4_ nanofibers combine to produce a material with strong DC and improved AC conductivity that performs well over a wide frequency range.

**Fig. 8:**
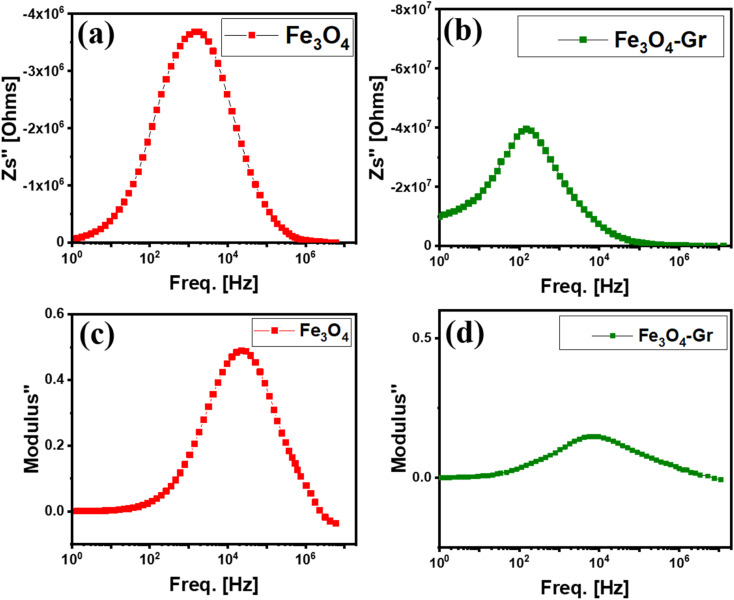
(a and b) Frequency-dependent imaginary part of impedance (*Z*′′) and (c and d) imaginary part of modulus (*M*′′) for pure Fe_3_O_4_ and Fe_3_O_4_-Gr nanofibers at room temperature.

At room temperature, there is a noticeable difference between pure Fe_3_O_4_ and Fe_3_O_4_-Gr nanofibers in the imaginary part of impedance, which indicates the reactive aspect of the material's reaction to an alternating current. In the case of pure Fe_3_O_4_ nanofibers, one single electrical response is detected at about 10^3^ Hz at room temperature, as shown in Fig. 8(a), suggesting a resonance point related to the intrinsic dielectric characteristics and magnetic polarization of the substance. Because the magnetite structure cannot form robust conductive networks, this reaction shows that the electrical activity of pure Fe_3_O_4_ nanofibers is mostly controlled by magnetic losses and has limited capacitive behavior.^34^ A slower charge transfer mechanism within the material is indicated by a higher frequency response, which is typical of magnetic materials with lower electrical conductivity. However, an electrical response is observed at a substantially lower frequency of approximately 10^2^ Hz in the Fe_3_O_4_-Gr nanofibers at room temperature, as shown in Fig. 8(b). The increased capacitive responsiveness and quicker polarization relaxation suggested by this shift toward lower frequencies are mainly caused by the addition of graphene to the nanofiber matrix. Because of its high electrical conductivity and vast surface area, graphene facilitates faster charge transfer and more effective electron mobility. The capacitive qualities of the nanofiber material are improved, and the total impedance is decreased by the conductive pathways created by the graphene sheets. This is reflected in the imaginary section of the impedance, demonstrating the significant impact of graphene on increasing charge storage capacity and reducing relaxation frequency.

A synergistic connection between the magnetic characteristics of pure Fe_3_O_4_ nanofibers and the extraordinary conductivity of graphene is highlighted by comparing the behavior of pure Fe_3_O_4_ and Fe_3_O_4_-Gr nanofibers. When graphene is added, a new conductive network is introduced, which dramatically changes the electrical properties of the nanofiber material, while pure Fe_3_O_4_ shows a primarily magnetic reaction. The nanofiber material that results from this interaction is more appropriate for applications that need both magnetic and conductive capabilities, such as EMI shielding materials or impedance-based sensors, since it lowers the impedance while shifting the relaxation processes to lower frequencies. The enhanced electrical response of Fe_3_O_4_-Gr nanofibers highlights the critical role that graphene plays in improving the conductive and dielectric qualities of nanofibers, allowing for more effective electrical behavior at lower frequencies.

At room temperature, pure Fe_3_O_4_ and Fe_3_O_4_-Gr nanofibers show interesting behavior in the imaginary part of the modulus, which measures a material's capacity to store and release energy under an applied electric field. At about 10^4^ Hz, a single electrical response for pure Fe_3_O_4_ nanofibers is observed, suggesting a comparatively sluggish relaxing process, as shown in Fig. 8(c). The observed high-frequency response can be interpreted as a reflection of the intrinsic dielectric constraints of Fe_3_O_4_; electroactive charge carriers encounter strong resistance, and polarization mechanisms lag behind lower-frequency electric fields. The imaginary modulus, therefore, stays high at lower frequencies, indicating that pure Fe_3_O_4_ releases electrical energy effectively at lower frequencies and instead depends mostly on its magnetic properties for energy dissipation.^35^

Fe_3_O_4_-Gr nanofibers also exhibit an electrical response of approximately 10^4^ Hz at room temperature, underlying relaxation dynamics are very different, as illustrated in Fig. 8(d). More effective charge mobility inside the nanofiber material is produced by the addition of graphene, even with the same peak frequency. The Fe_3_O_4_-Gr nanofiber material has a lower imaginary modulus over the frequency range because of the good polarization sensitivity and large surface area of graphene. This suggests that because graphene promotes faster charge redistribution, the nanofiber material performs better capacitively and has more effective energy storage capacities.^36^ Graphene-created conductive channels improve the overall dielectric response of the material by minimizing the energy losses that would otherwise occur in pure Fe_3_O_4_ nanofibers. Fe_3_O_4_ and graphene play complementary functions in determining the electrical characteristics of the nanofiber material, as demonstrated by a comparison of pure Fe_3_O_4_ and Fe_3_O_4_-Gr nanofibers at room temperature. Pure Fe_3_O_4_ nanofiber material has slower relaxation and greater energy dissipation at lower frequencies, which are mainly caused by its magnetic nature.^37^ However, the nanofiber material's capacity to interact with the electric field is improved by the addition of graphene, resulting in faster charge mobility and more effective polarization. Therefore, the Fe_3_O_4_-Gr nanofibers combine the magnetic loss processes of Fe_3_O_4_ with the improved conductivity of graphene to provide a nanofiber material that is more appropriate for applications that need both quick dielectric response and effective energy storage over a wider frequency range.

A vector network analyzer calculated the total shielding effectiveness created on the scattering parameters using the rectangular waveguide technique (x-band). The equations of SE_A_ SE_R_ and SE_T_ are as follows:^5^

1 SE_A =_ −10 log (1 − *A*_eff_),

2 SE_R_ = −10 log(1 − *R*),

3 SE_T =_ −10 log *T*.

**Fig. 9:**
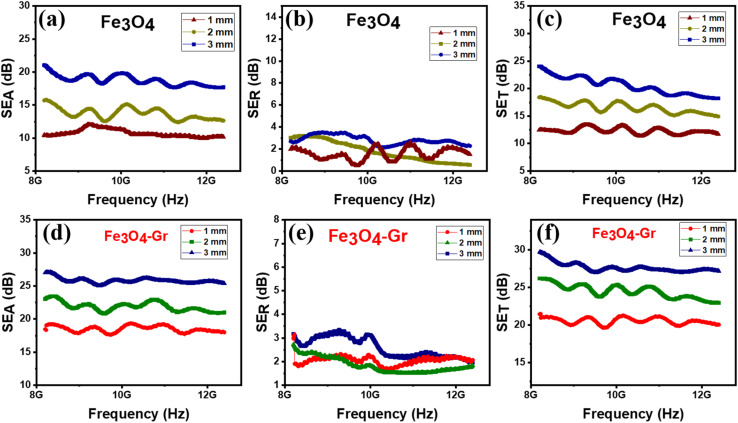
(a–c) Shielding effectiveness due to absorption (SE_A_), reflection (SE_R_) and total EMI shielding (SE_T_) for Fe_3_O_4_ and (d–f) for Fe_3_O_4_-Gr nanofibers in the X-band frequency range.

The electromagnetic interference (EMI) shielding performance of pure Fe_3_O_4_ and Fe_3_O_4_-Gr nanofibers exhibits a distinct comparison trend in the X-band when analyzing shielding efficiency owing to absorption (SE_A_), reflection (SE_R_), and total shielding (SE_T_). At 3 mm thickness, pure Fe_3_O_4_ nanofibers exhibit a clear absorption-dominant behavior with an SE_A_ of about 22 dB, as shown in Fig. 9(a), well above the reflection contribution (SE_R_ ∼3 dB), as shown in Fig. 9(b). At higher frequencies, pure Fe_3_O_4_ is susceptible to diminishing absorption efficiency, as evidenced by the total shielding effectiveness (SE_T_), which reaches ∼24 dB, as shown in Fig. 9(c); however, it shows a notable reduction with increasing frequency. The EMI shielding capabilities of pure Fe_3_O_4_ nanofibers are significantly improved when graphene is added. The Fe_3_O_4_-Gr nanofiber material exhibits higher shielding effectiveness owing to absorption SE_A_ ∼26 dB, as shown in Fig. 9(d), with a marginally enhanced SE_R_ of 3.5 dB, as shown in Fig. 9(e), and (SE_T_) of around 29 dB, as shown in Fig. 9(f). It is noteworthy that the SE_T_ of Fe_3_O_4_-Gr nanofibers does not change over the X-band frequency range, indicating the significance of graphene in providing constant shielding and electromagnetic absorption at high frequencies. This stability highlights the capacity of graphene to offer a conductive network that improves absorption without compromising shielding efficacy over a wide frequency range in contrast to the frequency-dependent drop shown in the pure Fe_3_O_4_ nanofibers.

SE_A_, SE_R_, and SE_T_ are found to decrease correspondingly with the material thickness for both pure Fe_3_O_4_ and Fe_3_O_4_-Gr nanofibers, from 3 mm to 1 mm (see Table 1). The decrease in total shielding for Fe_3_O_4_ nanofibers is more noticeable and indicates a decrease in material effectiveness as the thickness decreases. The lower thickness of Fe_3_O_4_-Gr nanofibers also results in a loss in shielding effectiveness; however, this drop is less pronounced when compared to pure Fe_3_O_4_, indicating the greater ability of the nanofiber material to maintain EMI shielding efficiency even in such situations. For absorption and frequency stability, this comparative behavior demonstrates the benefits of adding graphene to improve overall EMI shielding effectiveness at different thicknesses. Fe_3_O_4_ and graphene play comparable roles in EMI shielding, as demonstrated by the relative performance of these two materials. Better wave penetration and fewer initial reflection losses result from the material's impedance being closer to free space impedance at lower thicknesses (*e.g.*, 1 mm). Higher SE_R_ values are often the result of a stronger impedance mismatch caused by thicker materials, which also increases reflection at the air–material interface. Absorption and reflection mechanisms work together to control the shielding effectiveness of Fe_3_O_4_ and Fe_3_O_4_-Gr nanofibers. Electromagnetic waves interact with only the material's surface layer at higher frequencies because skin depth diminishes. The waves may partially pass through the 1 mm sample instead of being forcefully reflected owing to their relative thinness, which lowers the SE_R_ contribution. Graphene creates conductive channels in Fe_3_O_4_-Gr nanofibers that improve multiple scattering and absorption instead of just reflection. The majority of the incoming waves penetrate deeper and are dissipated rather than reflected, which lowers the SE_R_ for thinner samples. Because of changes in the dielectric constant, electrical conductivity, and wave–material interactions, the reflection efficiency does not necessarily increase in proportion to the frequency. For thinner samples, SE_R_ decreases because the enhanced conductivity at higher frequencies permits more energy to dissipate within the material rather than to be reflected at the surface. Although graphene improves conductivity and multi-reflection to produce better and more stable shielding, Fe_3_O_4_'s magnetic losses are crucial in absorbing electromagnetic radiation.^38^ The Fe_3_O_4_-Gr nanofiber material outperforms pure Fe_3_O_4_ owing to the synergy between the conductive network of graphene and the magnetic characteristics of Fe_3_O_4_. This is particularly true in applications where strong absorption and constant performance across a wide frequency range are required.

**Table 1:** Comparison of SE_A_, SE_R_ and SE_T_ for pure Fe_3_O_4_ and Fe_3_O_4_-Gr nanofibers at 3 mm thickness

Nanofibers material	Freq (GHz)	SE_A_ (dB)	SE_R_ (dB)	SE_T_ (dB)
Fe_3_O_4_	10.0	19.9	3.01	21.4
Fe_3_O_4_-Gr	10.0	26.01	2.4	28.51

## 4. Conclusion

This thorough investigation demonstrates notable improvements in the structural, electrical, and electromagnetic interference (EMI) shielding capabilities of Fe_3_O_4_ nanofibers with graphene placement. Fe_3_O_4_-fibers maintained their fibrous morphology, as demonstrated by FE-SEM analysis. The Fe_3_O_4_ spinel structure has been well-preserved in both nanofiber materials by XRD patterns. The bulk relaxation of both nanofiber materials is shown by impedance and modulus analysis at room temperature. The addition of graphene improved the electrical performance by lowering the total impedance and increasing the dielectric relaxation. Fe_3_O_4_-Gr nanofibers demonstrated the considerable influence of graphene on polarization and dielectric relaxation processes in terms of dielectric behavior, with two relaxation peaks, a decreased tangent loss across the frequency spectrum, and a greater dielectric constant. Graphene significantly enhanced conductivity at higher frequencies according to AC conductivity trends, which may have contributed to improved charge mobility. Most remarkably, because of the conductive network of graphene, the EMI shielding performance of Fe_3_O_4_-Gr nanofibers was higher than that of the pure Fe_3_O_4_ nanofiber material. Graphene is essential for sustaining high EMI shielding efficiency over a wide frequency range, as shown by the X-band's consistent total shielding effectiveness (SE_T_).

## Data availability

Supporting data that are related to this manuscript will be made available if mandatory.

## Conflicts of interest

The authors have no significant financial or non-financial benefits to disclose.
